# Relationship of loudness-dependent auditory evoked potentials with change-related cortical responses

**DOI:** 10.1371/journal.pone.0277153

**Published:** 2022-11-07

**Authors:** Kohei Fujita, Nobuyuki Takeuchi, Shunsuke Sugiyama, Koji Inui, Yuki Fujita, Ami Yamaba, Taeko Kamiya, Kousuke Kanemoto, Makoto Nishihara

**Affiliations:** 1 Neuropsychiatric Department, Aichi Medical University, Nagakute, Japan; 2 Department of Psychiatry, Okazaki City Hospital, Okazaki, Japan; 3 Department of Psychiatry and Psychotherapy, Gifu University, Gifu, Japan; 4 Department of Integrative Physiology, National Institute for Physiological Sciences, Okazaki, Japan; 5 Department of Functioning and Disability, Institute for Developmental Research, Aichi Developmental Disability Center, Kasugai, Japan; 6 Central clinical laboratory, Aichi medical university Hospital, Nagakute, Japan; 7 Department of Psychiatry, Kamibayashi memorial Hospital, Ichinomiya, Japan; 8 Multidisciplinary Pain Center, Aichi Medical University, Nagakute, Japan; Georgia State University, UNITED STATES

## Abstract

Previous studies have suggested that change-related cortical responses are phenomena similar to the onset response and could be applied to the loudness dependence of auditory evoked potential (LDAEP) paradigm. In the present study, we examined the relationship between LDAEP and the change-related response using electroencephalography findings in 50 healthy subjects. There were five conditions (55, 65, 75, 85, and 95 dB) for LDAEP and five similar conditions (abrupt sound pressure increase from 70 to 75, 80, 85, 90, and 95 dB) for the change-related response. Both the onset and abrupt sound pressure increase evoked a triphasic response with peaks at approximately 50 (P50), 100 (N100), and 200 (P200) ms. We calculated the peak-to-peak amplitudes for P50/N100 and N100/P200. Medians and slopes for P50/N100 and N100/P200 amplitudes were calculated and compared between the two measures. Results revealed a significant correlation for both the slope and median for P50/N100 (*r* = 0.36, 0.37, p = 1.0 × 10^−2^, 7.9 × 10^−3^), N100/P200 (*r* = 0.40, 0.34, p = 4.0 × 10^−3^, 1.6 × 10^−2^), and P50/N100/P200 (*r* = 0.36, 0.35, p = 1.0 × 10^−2^, 1.3 × 10^−2^). These results suggested that the change-related response and LDAEP shared generation mechanisms at least partially.

## Introduction

Although not precisely defined, the loudness dependence of auditory evoked potentials (LDAEP) is an electrophysiological measure used to evaluate changes in the amplitude of evoked potentials among several sound levels (about 4, 5, or 6) [1]. The amplitude increases with an increase in stimulus intensity but reaches a plateau at high intensities such as >90 dB. It is believed that this phenomenon reflects inhibitory mechanisms that protect against excessive sensory information. Studies have shown that the LDAEP slope is related to several mental disorders, including major depressive [2], bipolar [3], generalized anxiety [4], and obsessive-compulsive [5] disorders. However, in a detailed review, Roser et al. pointed out that in major depression, no differences in LDAEP were found among the non-medicated, medicated, and normal control groups [6]. Furthermore, LDAEP behaves differently in different subtypes of depression, such as atypical depression and melancholic depression [7]; therefore, it requires careful interpretation. The slope is also associated with temporal summation of nociceptive fiber-evoked responses [8] and several pain-related disorders in which sensitization of the central nervous system is assumed, including dysmenorrhea [9], migraine [10], and fibromyalgia [11]. Previous studies, including meta-analyses, have demonstrated that LDAEP may be a promising predictor of responsiveness to selective serotonin reuptake inhibitor (SSRI) treatments for depression and generalized anxiety disorder and may have potential clinical applications [12–14]. These studies are based on the assumption that LDAEP is associated with central serotonergic function. In animal studies, SSRIs are observed to have decreased the slope of LDAEP when the release of serotonin in the synaptic cleft by SSRIs in the auditory cortex was increased [15], and LDAEP changed with changes in the firing rate of serotonin neurons in the dorsal raphe nucleus by serotonergic pharmacological manipulation [16]. However, the evidence for the relationship between serotonin and LDAEP in humans is inconsistent. For example, changes in LDAEP in humans are presumed to be affected by increased synaptic serotonin concentrations following acute SSRI administration, but the results are inconsistent [6, 17, 18]. Serotonin gene polymorphisms and sex differences have been suggested as possible reasons for this, but they are still unclear [19, 20]. Furthermore, LDAEP has been implicated in serotonin and other neurotransmitters, such as dopamine, noradrenaline, and glutamate [6, 19, 21, 22].

Change-related cortical responses are specifically elicited by abrupt changes in a continuous sensory stimulus and can be clearly recorded without attention by the participant [23–26]. The immediate detection of changes to adapt to an ever-changing environment is an important ability to survive. The change-related response is induced by an abrupt change in sound features [23, 27], and the magnitude of the response varies logarithmically with the difference in physical quantity between the preceding and current stimuli [23]. In other words, the amplitude of the change-related response depends on the magnitude of the change [23, 28]. This response exhibits good test–retest reliability [28, 29] with short interstimulus intervals and has been observed in auditory [23], somatosensory [29], and visual [30] systems. In other words, the change-related response reflects the fundamental information processing that is common across sensory modalities. Moreover, it has been reported that the amplitude of change-related potentials evoked by a transient decrease in binaural correlation is reduced in schizophrenia [31]. Therefore, it is anticipated that change-related responses can be utilized for clarifying the mechanism of schizophrenia.

We have proposed that the onset response of auditory evoked potentials (AEP) is a form of the change-related response because the latency and amplitude of the two measures exhibit similar behaviors in response to sound pressure changes [25] and their neural origins are similar [32, 33]. A new method based on the change-related response via electroencephalograms (EEGs) has been proposed for use in physiological tests such as paired-pulse suppression or prepulse inhibition as it results in high reliability and short inspection time [34, 35]. Because LDAEP is a measure of the onset response, it is important to understand whether the change-related response elicited by sound pressure changes exhibits a similar pattern to LDAEP. If this is the case, change-related responses could serve as an electrophysiological tool to evaluate certain diseases. Therefore, in the present study, we recorded LDAEP and the change-related response and investigated the correlation between them.

## Methods

### Participants

Our study subjects consisted of 50 volunteers (20 women, 30 men; mean age of 37.0 years) who had normal hearing (based on self-report), had no history of mental or neurological disorders or substance abuse in the most recent 5 years, and were free of medication at the time of testing. The study protocol was designed according to the Declaration of Helsinki and approved in advance by the Ethics Committee of the National Institute for Physiological Sciences, Okazaki, Japan. Each subject provided written consent before participation.

### Auditory stimuli

**Fig 1:**
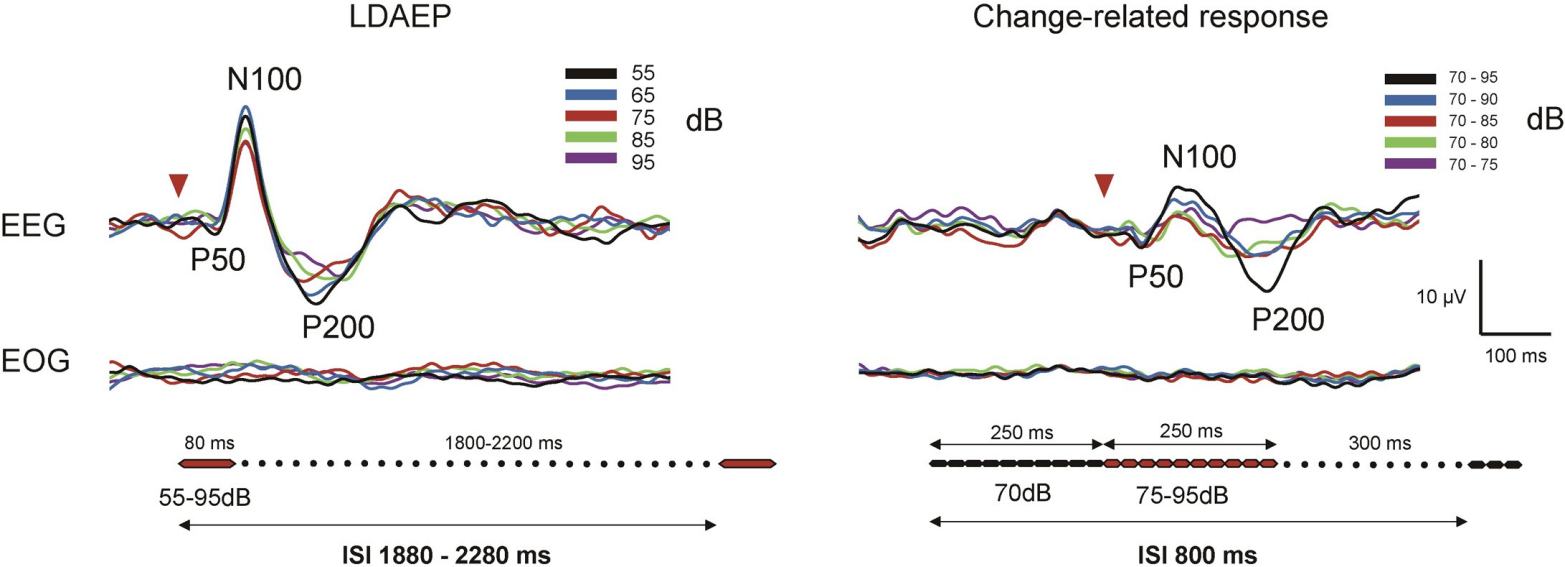
Waveforms of a representative subject. Both the onset and abrupt sound pressure increase evoked a triphasic response with peaks at approximately 50 (P50), 100 (N100), and 200 (P200) ms, with peak amplitudes measured in the time windows of 30–80, 80–150, and 150–280 ms, respectively. The EOG does not affect evoked potentials for either LDAEP or change-related responses. LDAEP, loudness-dependent auditory evoked potentials; ISI, interstimulus interval.

Auditory stimuli were created by a personal computer (Panasonic CF-RZ6, Windows XP 32 bit) and presented binaurally via earpieces (E-A-Rtone 3A, Aero Company, Indianapolis, IN). For LDAEP, an 80 ms pure tone at 800 Hz (rise/fall, 10 ms to avoid undesired edge) was presented at five different sound pressure levels (55, 65, 75, 85, and 95 dB SPL). The interstimulus interval was randomized between 1800 and 2200 ms (Fig 1A). Tones of five intensities were intermixed and presented randomly without restriction.

For the change-related response, a train of 25 ms pure tones at 800 Hz (rise/fall, 5 ms), i.e., 40-Hz amplitude modulated sound, was used. Sound stimuli had a total duration of 500 ms consisting of 20 repeats of a pure tone with a sound pressure of 70 dB SPL. To elicit change-related responses, the sound pressure of the pure tones after 250 ms was increased to 75, 80, 85, 90, or 95 dB. In addition to these five conditions, a control condition was included without such a sound pressure change. The stimulus onset asynchrony was 800 ms, which resulted in an intertrial interval of 300 ms (Fig 1B). Tones of six intensities were intermixed and presented randomly without restriction. Calibration of the sound level was performed using a sound level meter (Rion NL-32) for each experiment.

### Recording procedures

Each subject sat in a comfortable chair in a quiet electrically shielded room and watched a silent movie and was instructed to ignore sound stimuli. An exploring electrode was placed at a midline central site referenced to as the linked mastoids [36]. A pair of electrodes were placed on the supra- and infra-orbits of the left eye and used for recording electrooculograms (EOGs). The EEG artifact rejection was set to 100 μV, and if the simultaneously recording EOG signals were greater than 100 μV, the epoch was removed. The impedance for all electrodes was maintained at <5 kΩ. AEPs were recorded at a sampling rate of 1000 Hz with a band-pass filter of 0.1–100 Hz (Neuropack MEB-2300, Nihon Kohden, Tokyo). The baseline was set at 100 ms before the sound onset and 100 ms before the change onset for LDAEP and the change-related response, respectively. At least 100 epochs for LDAEP and 120 epochs for the change-related response were averaged.

### Analysis

The AEP components were analyzed after applying a digital filter of 0.98–35.2 Hz digital after epoching at zero phase, 24 dB/octave. Both the onset and abrupt sound pressure increase evoked a triphasic response with peaks at approximately 50 (P50), 100 (N100), and 200 (P200) ms, with peak amplitudes measured in the time windows of 30–80, 80–150, and 150–280 ms, respectively. Peak-to-peak amplitudes were calculated for P50/N100 and N100/P200. P50/N100/P200 was calculated as the sum of the amplitudes of P50/N100 and N100/P200. Such a procedure to measure peak-to-peak amplitudes minimizes problems related to baseline shift [24]. The change-related responses for each condition were obtained by subtracting waveforms for the control stimulus from those for five stimuli with changes. LDAEP is generally analyzed as the slope of the amplitude/stimulus intensity function among five sound pressure levels. The slope was calculated as (1) a linear regression line of the amplitude of the five points (linear slope) or (2) a median of the slopes between all 10 pairs among the five conditions (N1/P2 55 dB and N1/P2 65 dB, N1/P2 55 dB and N1/P2 75 dB, N1/P2 55 dB and N1/P2 85 dB and so forth) (median slope) [37]. The slope was expressed as the amplitude change per stimulus intensity difference (μV/10 dB). The absolute value of correlation coefficients was considered to indicate a weak correlation when 0.1 < *r* < 0.3, moderate when 0.3 ≤ *r* < 0.5, and strong when *r* ≥ 0.5 [36]. We performed partial correlation analysis using the amplitude of the stimulus at 55 dB as a control factor to rule out the possibility that LDAEP and change-dependent responses are associated because of overall larger ERPs in some participants. For this linear slope and median slope, we conducted an additional subanalysis grouped by sex. Sex-specific relationships between the slopes of LDAEP and change-related responses were determined via Pearson’s correlation coefficient and statistically compared using “cocor” http://comparingcorrelations.org/ based on a modification of Fisher’s Z procedure [38].

## Results

**Fig 2:**
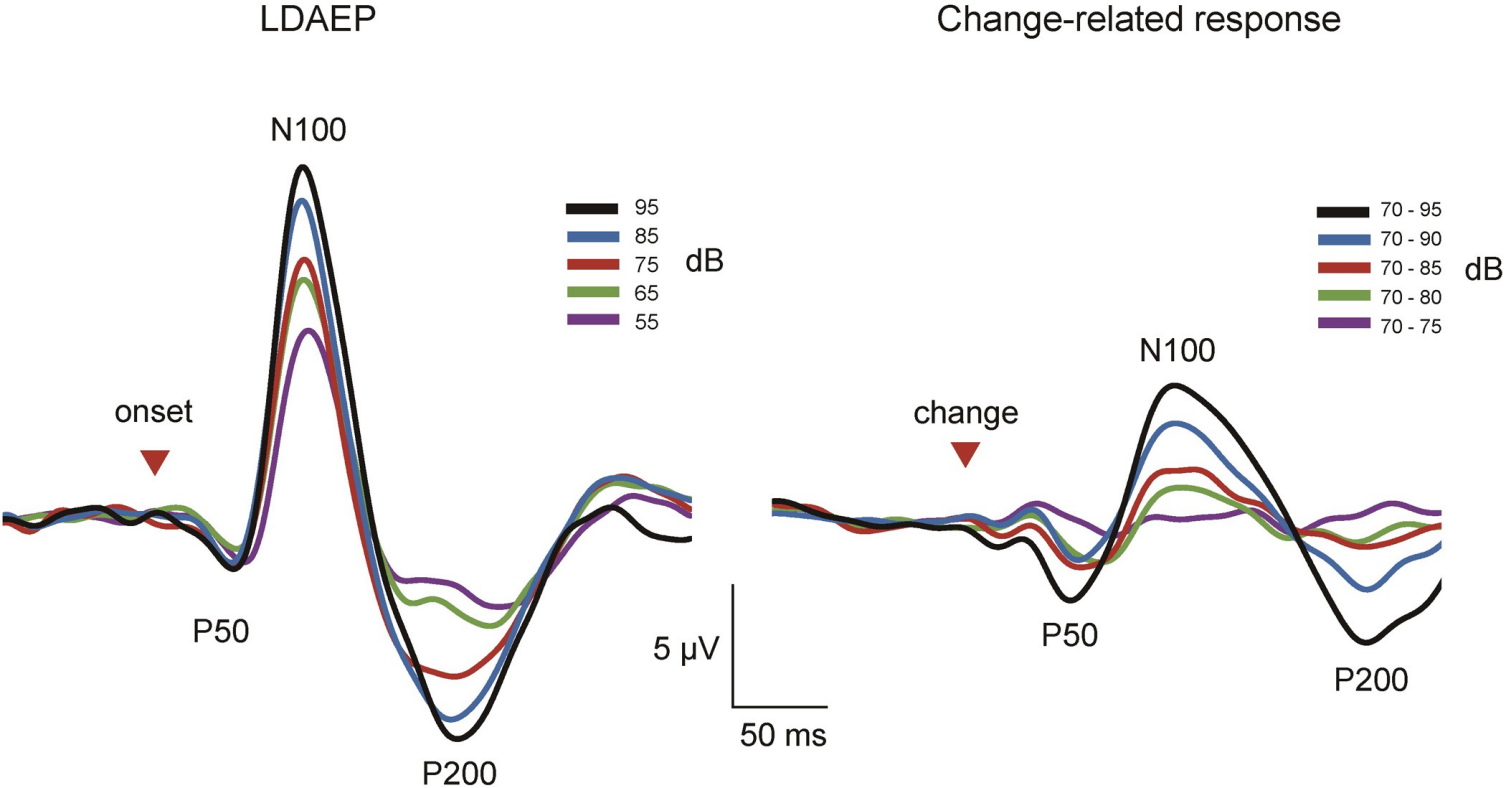
Grand-averaged waveforms of the onset and change-related responses. Grand-averaged waveforms of the onset responses (A) and change-related responses (B) for each condition. Red arrowheads indicate the sound (A) and change (B) onsets.

**Fig 3:**
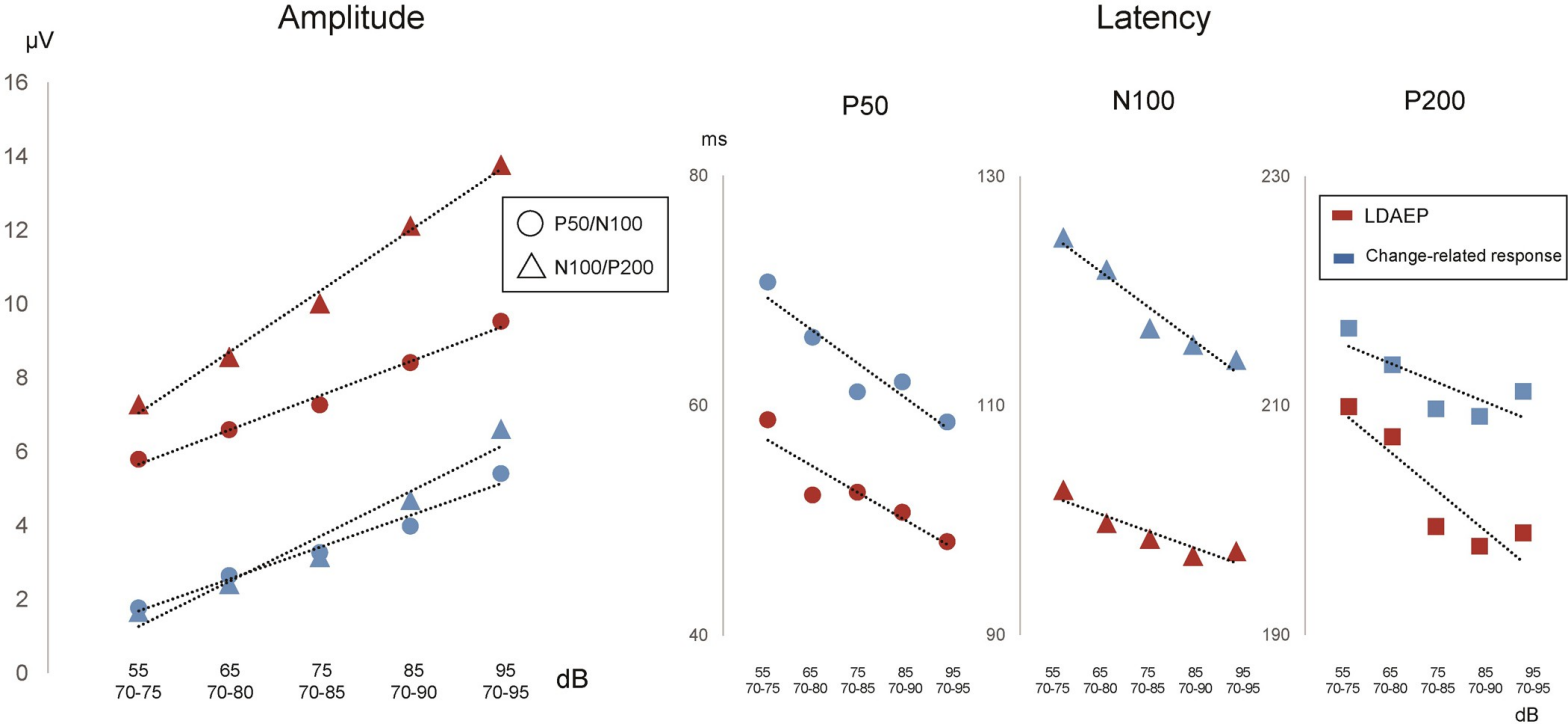
Amplitude and latency for LDAEP and the change-related response. LDAEP and the change-related response are shown aligned. On the right are the P50/N100 and N100/P200 amplitudes corresponding to sound pressure and sound pressure changes, respectively, and on the left are the P50, N100, and P200 latencies corresponding to sound pressure and sound pressure changes.

**Fig 4:**
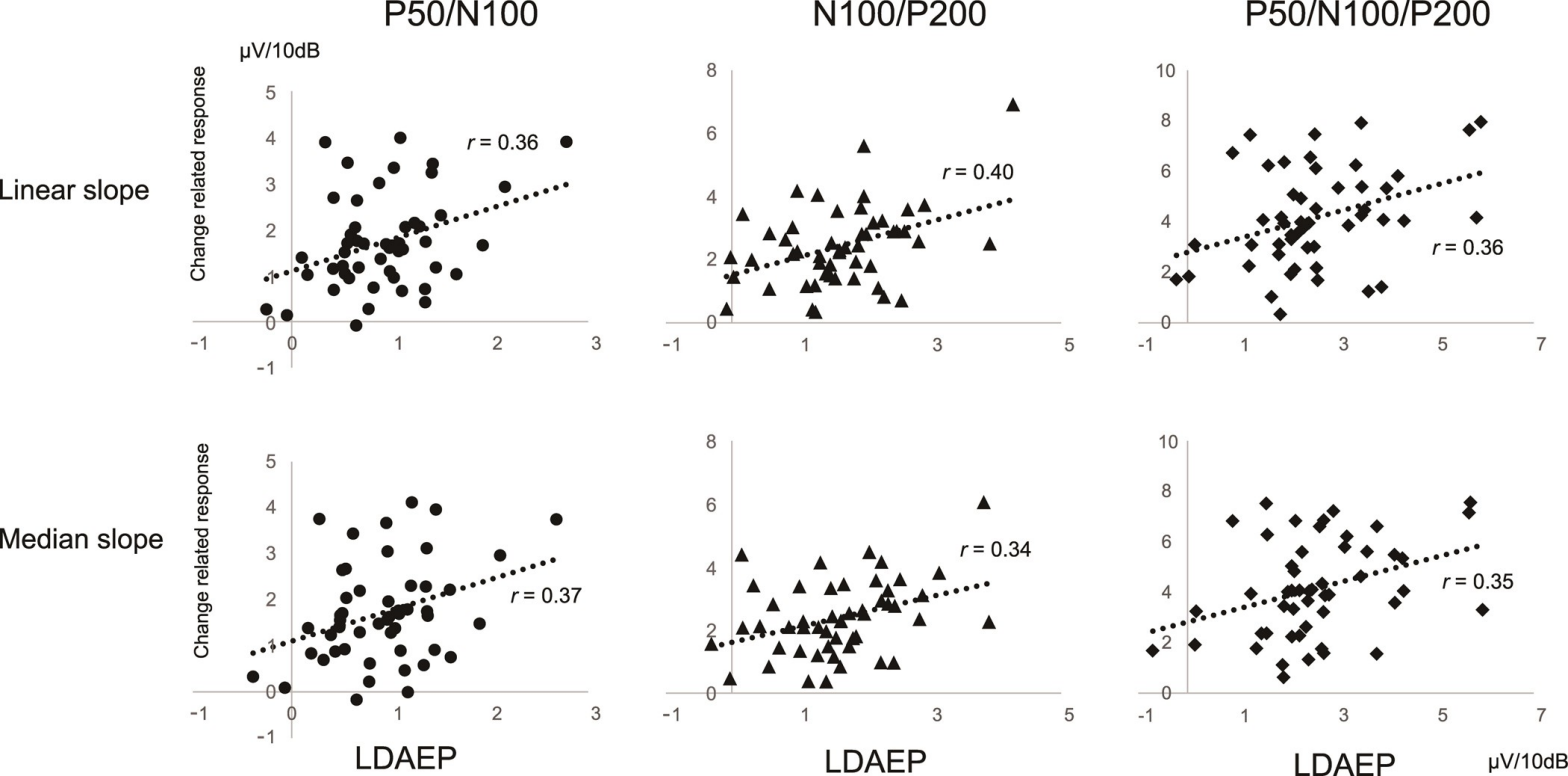
Correlation of the amplitude slope between LDAEP and the change-related response. Correlations between LDAEP and the change-related response in P50/N100, N100/P200, and P50/N100/P200 are shown in the linear slope and median slope.

Fig 1 shows the representative AEP waveforms of a single subject. Table 1 lists the mean slope across subjects. Grand-averaged waveforms are presented in Fig 2. The amplitude of P50/N100 and N100/P200 for both LDAEP and the change-related response became larger and the latency of all components became shorter with an increase in sound pressure and in the degree of sound pressure change, respectively (Fig 3). A correlation was found between LDAEP and the change-related response for all slopes for P50/N100 (*r* = 0.36, 0.37, p = 1.0 × 10^−2^, 7.9 × 10^−3^), N100/P200 (*r* = 0.40, 0.34, p = 4.0 × 10^−3^, 1.6 × 10^−2^), and P50/N100/P200 (*r* = 0.36, 0.35, p = 1.0 × 10^−2^, 1.3 × 10^−2^) (Fig 4). Partial correlations between LDAEP and change-related responses, controlling for the amplitude of the stimulus at 55 dB as the baseline ERP strength, were significant across all slopes for P50/N100 (r = 0.38, 0.40, p = 7.0 × 10^−3^, 4.0 × 10^−3^), N100/P200 (r = 0.48, 0.40, p = 4.0 × 10^−4^, 4.0 × 10^−3^), and P50/N100/P200 (r = 0.44, 0.42, p = 3.0 × 10^−3^, 2.0 × 10^−3^). An additional subanalysis grouped by sex was conducted. For men, a significant correlation was obtained for both slopes for P50/N100 (*r* = 0.40, 0.40, p = 2.9 × 10^−2^, 3.0 × 10^−2^) but not for N100/P200 (*r* = 0.16, 0.09, p = 0.41, 0.65), and for women, no significant correlation was obtained for both slopes for P50/N100 (*r* = 0.26, 0.22, p = 0.28, 0.36), but a significant correlation was obtained for N100/P200 (*r* = 0.67, 0.68, p = 1.3 × 10^−3^, 1.1 × 10^−3^). In the linear slope analysis, the difference in correlation coefficients between LDAEP and the change-related response between women and men was not significant at p = 0.60 for P50/N100 but was significant at p = 0.04 for N100/P200. We performed the same analysis for the median slope, with p = 0.28 for P50/N100 and p = 0.03 for N100/P200 (S1 Fig).

**Table 1:** Linear and median slopes. Values are shown as the mean (SD).

		LDAEP					Change-related response				
**Stimulus (dB)**		55	65	75	85	95	70–75	70–80	70–85	70–90	70–95
**Liner (μV/10db)**	P50/N100	0.9 (0.5)					1.7 (1.0)				
	N100/P200	1.7 (0.9)					2.4 (1.3)				
	P50/N100/P200	2.6 (1.3)					4.2 (1.9)				
**Median (μV/10db)**	P50/N100	0.9 (0.5)					1.7 (1.0)				
	N100/P200	1.6 (0.9)					2.4 (1.2)				
	P50/N100/P200	2.6 (1.3)					4.1 (1.9)				
**Amplitude (μV)**	P50/N100	5.8 (2.5)	6.6 (2.7)	7.3 (2.8)	8.4 (3.4)	9.5 (3.4)	1.8 (1.4)	2.6 (1.5)	3.3 (2.0)	4.0 (2.1)	5.4 (2.7)
	N100/P200	7.3 (3.2)	8.6 (3.5)	10.0 (3.6)	12.1 (4.4)	13.8 (4.4)	1.7 (1.5)	2.4 (1.3)	3.1 (1.7)	4.7 (1.8)	6.6 (3.0)
	P50/N100/P200	13.1 (5.4)	15.2 (5.9)	17.3 (6.1)	20.5 (7.4)	23.3 (7.2)	3.4 (2.5)	5.0 (2.4)	6.4 (3.3)	8.7 (3.6)	12.0 (5.1)
**Latency (ms)**	P50	58.8 (9.3)	52.2 (11.3)	52.5 (9.8)	50.7 (9.9)	48.2 (11.0)	70.8 (14.7)	66.0 (14.9)	61.2 (15.1)	62.1 (14.1)	58.6 (14.2)
	N100	102.7 (10.0)	99.8 (9.6)	98.4 (7.7)	96.9 (7.6)	97.3 (7.3)	124.7 (22.3)	121.9 (17.6)	116.7 (18.6)	115.3 (15.8)	114.0 (14.3)
	P200	209.9 (27.5)	207.8 (27.9)	199.5 (23.5)	197.7 (19.1)	198.9 (26.8)	216.8 (30.1)	213.6 (27.3)	209.7 (28.3)	209.1 (24.8)	211.3 (24.7)

## Discussion

The amplitude of P50/N100 and N100/P200 positively correlated between LDAEP and the change-related response in two different calculation methods, i.e., linear slope and median slope. To the best of our knowledge, this is the first study in which the slopes of the change-related response were calculated. The results of this study suggested that change-related responses can be used as an electrophysiological tool as well as LDAEP [6].

### Similarity and dissimilarity between the onset and change-related responses

Some similar characteristics exist between the onset and change-related responses. Previous studies have shown that both responses exhibit a clear triphasic configuration with the N100 component that increases in amplitude and shortens in latency with a greater sound intensity [25, 39–41]. As mentioned in those studies, the latency became shorter and responses became greater with the increase in the sound pressure for both LDAEP and the change-related response in the present study (Fig 3).

The onset and change-related responses are also characterized by the fact that the responses are determined by not only sound pressure but also the preceding sound duration. The amplitude and latency of N100 of the onset response are determined by the interstimulus interval [42–44], whereas those of the change-related response are determined by the length of the preceding sound before the change onset [23, 24, 26]. This is because both responses are dependent on a comparison between the present and preceding sensory status, the process of which involves sensory memory [23, 25]. The length of the sound before the change onset to store information in memory is important for the change-related response, whereas the length of the blank before the sound onset for the decay of memory of the previous sound is important for the onset response [23, 27, 33]. Therefore, although the two measures share some mechanisms such as comparison processes, they differ in terms of which memory process is important, storage or decay, which results in different ISIs needed. If short ISIs are used for LDAEP, the memory trace does not decay sufficiently to evoke change-related components, and the response would no longer exhibit clear sound pressure dependence. At least within a certain range, the amplitude of the change-related response depends on the magnitude of changes but not the strength of the stimulus itself [23, 25, 45]. Therefore, in exchange for the time it takes to measure, LDAEP has a merit that the response is greater in amplitude than the change-related response elicited with short ISIs as demonstrated in the present study. This indicates better SN ratios and thus higher reliability of obtained data.

### P50/N100 component

Although LDAEP studies generally analyze only N100/P200, we included P50/N100 in the present study. Studies have reported that the P50 component was related to the cholinergic brain stem function [46], arousal level [47], mild cognitive impairment [48], and age-related changes [49]. Moreover, previous studies have shown that P50/N100 and N100/P200 exhibit different behaviors in a suppression paradigm [34, 35]. Therefore, P50/N100 can be a novel physiological indicator that reflects different aspects of sound pressure- or sound pressure change-related cortical responses. In a subanalysis by sex, we detected a significant correlation for men at P50/N100, but not for women. By contrast, N100/P200 exhibited a significant correlation for women, but not for men. For LDAEP, a steeper N100/P200 slope has been reported for women [20], and the available components may differ by sex. This suggests that the P50/N100 component reflects a different physiological aspect than N100/P200, and it would be meaningful to include a P50/N100 component in the LDAEP analysis, especially when sex differences are considered. However, the number of subjects in this subanalysis was limited, and further studies are required. One problem with measuring P50 is its lower reliability than the N100/P200 component [34, 35]. This problem can be addressed using peak-to-peak amplitudes because P50 is sensitive to baseline shift. Using noise bursts, complex tones combining multiple frequencies or click sounds are effective in improving the signal-to-noise ratio of P50. Therefore, in future studies one should consider using one of these sounds to induce a clearer P50.

### Clinical implications

LDAEP is not a specific or sensitive biomarker for any psychiatric disorder, but it can serve as a promising tool for the prediction of antidepressant treatment response in patients with depression [6, 12]. Additionally, its recording takes more than 30 min. As patients with mental disorders often have difficulties sitting still for a long time, it is important to shorten the measurement time. Unlike the onset response, the ISI is not critical for the change-related response. The change-related response that takes approximately 15 min is relatively short compared with conventional methods. Further investigations are required to evaluate the relationship between the change-related response and, for example, the responsiveness to SSRIs in patients with major depressive disorders or generalized anxiety disorders.

## Limitations

This study had some limitations. First, participants confirmed that they had no hearing impairment only via self-reporting, no actual hearing test was performed. Second, we used only one EEG derivation. Although a single electrode is a common method to measure LDAEP, there is no study using dipole analyses for measuring the slopes of the change-related response. Finally, the change-related response tends to have relatively high SD values. In some subjects, deflections were low in amplitude, which resulted in unclear peaks, but the measurement was performed based on the definition (e.g., P50 components were measured with maximum amplitude between 30 and 80 ms). To establish this approach as a clinical tool, future studies on both healthy controls and patients with related mental disorders should be conducted.

## Supporting information

S1 Fig Sex differences in LDAEP and the change-related response in P50/N100 and N100/P200. Differences in correlation coefficients between LDAEP and the change-related response by are shown for the P50/N100 and N100/P200 components.
(TIF)

## References

[1] Hegerl U, Juckel G. Intensity dependence of auditory evoked potentials as an indicator of central serotonergic neurotransmission: a new hypothesis. Biol Psychiatry 1993;33: 173–187. doi: 10.1016/0006-3223(93)90137-3 8383545

[2] Leuchter AF, Cook IA, Hunter A, Korb A. Use of clinical neurophysiology for the selection of medication in the treatment of major depressive disorder: the state of the evidence. Clin EEG Neurosci 2009;40: 78–83. doi: 10.1177/155005940904000207 19534301

[3] Lee KS, Park YM, Lee SH. Serotonergic dysfunction in patients with bipolar disorder assessed by the loudness dependence of the auditory evoked potential. Psychiatry Investig 2012;9: 298–306. doi: 10.4306/pi.2012.9.3.298 22993531 PMC3440481

[4] Park YM, Kim DW, Kim S, Im CH, Lee SH. The loudness dependence of the auditory evoked potential (LDAEP) as a predictor of the response to escitalopram in patients with generalized anxiety disorder. Psychopharmacology 2011;213: 625–632. doi: 10.1007/s00213-010-2061-y 21057773

[5] Mavrogiorgou P, Enzi B, Steinmann S, Mulert C, Juckel G. Relationship between neuroanatomical and serotonergic hypotheses of obsessive-compulsive disorder. The Journal of Clinical Psychiatry 2018. doi: 10.4088/jcp.17m11811 30326190

[6] Roser P, Kawohl W, Juckel G. The loudness dependence of auditory evoked potentials as an electrophysiological marker of central serotonergic neurotransmission: implications for clinical psychiatry and psychopharmacotherapy. Handbook of Behavioral Neuroscience 2020; 361–374. doi: 10.1016/b978-0-444-64125-0.00020-7

[7] Lee S-H, Park Y-C, Yoon S, Kim J-I, Hahn SW. Clinical implications of loudness dependence of auditory evoked potentials in patients with atypical depression. Prog Neuropsychopharmacol Biol Psychiatry. 2014;54: 7–12. doi: 10.1016/j.pnpbp.2014.05.010 24865151

[8] Uhl I, Krumova EK, Regeniter S, Bär KJ, Norra C, Richter H, et al. Association between wind-up ratio and central serotonergic function in healthy subjects and depressed patients. Neurosci Lett 2011;504: 176–180. doi: 10.1016/j.neulet.2011.09.033 21964385

[9] Zhang B, Xu Y, He W, Wang J, Chai H, Shen C, et al. Intensity dependence of auditory evoked potentials in primary dysmenorrhea. J Pain 2017;18: 1324–1332. doi: 10.1016/j.jpain.2017.06.009 28694148

[10] Ambrosini A, Kisialiou A, Coppola G, Finos L, Magis D, Pierelli F, et al. Visual and auditory cortical evoked potentials in interictal episodic migraine: an audit on 624 patients from three centres. Cephalalgia 2017;37: 1126–1134. doi: 10.1177/0333102416665224 27582121

[11] Carrillo-de-la-Peña MT, Vallet M, Pérez MI, Gómez-Perretta C. Intensity dependence of auditory-evoked cortical potentials in fibromyalgia patients: a test of the generalized hypervigilance hypothesis. J Pain 2006;7: 480–487. doi: 10.1016/j.jpain.2006.01.452 16814687

[12] Yoon S, Kim Y, Lee SH. Does the loudness dependence of auditory evoked potential predict response to selective serotonin reuptake inhibitors?: A meta-analysis. Clin Psychopharmacol Neurosci 2021;19: 254–261. doi: 10.9758/cpn.2021.19.2.254 33888654 PMC8077049

[13] Gallinat J, Bottlender R, Juckel G, Munke-Puchner A, Stotz G, Kuss HJ, et al. The loudness dependency of the auditory evoked N1/P2-component as a predictor of the acute SSRI response in depression. Psychopharmacology 2000;148: 404–411. doi: 10.1007/s002130050070 10928314

[14] Jaworska N, Blondeau C, Tessier P, Norris S, Fusee W, Blier P, et al. Response prediction to antidepressants using scalp and source-localized loudness dependence of auditory evoked potential (LDAEP) slopes. Prog Neuropsychopharmacol Biol Psychiatry 2013;44: 100–107. doi: 10.1016/j.pnpbp.2013.01.012 23360662 PMC3654010

[15] Wutzler A, Winter C, Kitzrow W, Uhl I, Wolf RJ, Heinz A, et al. Loudness dependence of auditory evoked potentials as indicator of central serotonergic neurotransmission: simultaneous electrophysiological recordings and in vivo microdialysis in the rat primary auditory cortex. Neuropsychopharmacology 2008;33: 3176–3181. doi: 10.1038/npp.2008.42 18463629

[16] Juckel G, Hegerl U, Molnár M, Csépe V, Karmos G. Auditory evoked potentials reflect serotonergic neuronal activity—a study in behaving cats administered drugs acting on 5-HT1A autoreceptors in the dorsal raphe nucleus. Neuropsychopharmacology 1999;21: 710–716. doi: 10.1016/S0893-133X(99)00074-3 10633476

[17] Nathan PJ, Segrave R, Phan KL, O’Neill B, Croft RJ. Direct evidence that acutely enhancing serotonin with the selective serotonin reuptake inhibitor citalopram modulates the loudness dependence of the auditory evoked potential (LDAEP) marker of central serotonin function. Hum Psychopharmacol 2006;21: 47–52. doi: 10.1002/hup.740 16317803

[18] Oliva J, Leung S, Croft RJ, O’Neill BV, O’Kane J, Stout J, et al. The loudness dependence auditory evoked potential is insensitive to acute changes in serotonergic and noradrenergic neurotransmission. Hum Psychopharmacol 2010;25: 423–427. doi: 10.1002/hup.1133 20589921

[19] O’Neill BV, Croft RJ, Nathan PJ. The loudness dependence of the auditory evoked potential (LDAEP) as an in vivo biomarker of central serotonergic function in humans: rationale, evaluation and review of findings. Hum Psychopharmacol. 2008;23: 355–370. doi: 10.1002/hup.940 18421800

[20] Oliva JL, Leung S, Croft RJ, O’Neill BV, Stout JC, Nathan PJ. Evidence for sex differences in the loudness dependence of the auditory evoked potential in humans. Hum Psychopharmacol 2011;26: 172–176. doi: 10.1002/hup.1187 21455974

[21] Kenemans JL, Kähkönen S. How human electrophysiology informs psychopharmacology: from bottom-up driven processing to top-down control. Neuropsychopharmacology 2011;36: 26–51. doi: 10.1038/npp.2010.157 20927044 PMC3055493

[22] Conti F, Minelli A, DeBiasi S, Melone M. Neuronal and glial localization of NMDA receptors in the cerebral cortex. Mol Neurobiol 1997;14: 1–18. doi: 10.1007/BF02740618 9170098

[23] Inui K, Urakawa T, Yamashiro K, Otsuru N, Nishihara M, Takeshima Y, et al. Non-linear laws of echoic memory and auditory change detection in humans. BMC Neurosci 2010;11: 80. doi: 10.1186/1471-2202-11-80 20598152 PMC2904354

[24] Inui K, Urakawa T, Yamashiro K, Otsuru N, Takeshima Y, Nishihara M, et al. Echoic memory of a single pure tone indexed by change-related brain activity. BMC Neurosci 2010;11: 135. doi: 10.1186/1471-2202-11-135 20961454 PMC2978218

[25] Nishihara M, Inui K, Motomura E, Otsuru N, Ushida T, Kakigi R. Auditory N1 as a change-related automatic response. Neurosci Res 2011;71: 145–148. doi: 10.1016/j.neures.2011.07.004 21787811

[26] Nishihara M, Inui K, Morita T, Kodaira M, Mochizuki H, Otsuru N, et al. Echoic memory: investigation of its temporal resolution by auditory offset cortical responses. PLOS ONE 2014;9: e106553. doi: 10.1371/journal.pone.0106553 25170608 PMC4149571

[27] Ohoyama K, Motomura E, Inui K, Nishihara M, Otsuru N, Oi M, et al. Memory-based pre-attentive auditory N1 elicited by sound movement. Neurosci Res 2012;73: 248–251. doi: 10.1016/j.neures.2012.04.003 22525281

[28] Inui K, Tsuruhara A, Nakagawa K, Nishihara M, Kodaira M, Motomura E, et al. Prepulse inhibition of change-related P50m no correlation with P50m gating. Springerplus 2013;2: 588. doi: 10.1186/2193-1801-2-588 24255871 PMC3825222

[29] Otsuru N, Tsuruhara A, Motomura E, Tanii H, Nishihara M, Inui K, et al. Effects of acute nicotine on auditory change-related cortical responses. Psychopharmacology 2012;224: 327–335. doi: 10.1007/s00213-012-2757-2 22707251

[30] Urakawa T, Inui K, Yamashiro K, Tanaka E, Kakigi R. Cortical dynamics of visual change detection based on sensory memory. Neuroimage 2010;52: 302–308. doi: 10.1016/j.neuroimage.2010.03.071 20362678

[31] Zheng Y, Liu L, Li R, Wu Z, Chen L, Li J, et al. Impaired interaural correlation processing in people with schizophrenia. Eur J Neurosci 2021;54: 6646–6662. doi: 10.1111/ejn.15449 34494695

[32] Yamashiro K, Inui K, Otsuru N, Kida T, Kakigi R. Automatic auditory off-response in humans: an MEG study. Eur J Neurosci 2009;30: 125–131. doi: 10.1111/j.1460-9568.2009.06790.x 19519639

[33] Yamashiro K, Inui K, Otsuru N, Kakigi R. Change-related responses in the human auditory cortex: an MEG study. Psychophysiology 2011;48: 23–30. doi: 10.1111/j.1469-8986.2010.01038.x 20525009

[34] Takeuchi N, Fujita K, Kinukawa T, Sugiyama S, Kanemoto K, Nishihara M, et al. Test–retest reliability of paired pulse suppression paradigm using auditory change-related response. J Neurosci Methods 2021;352: 109087. doi: 10.1016/j.jneumeth.2021.109087 33508410

[35] Takeuchi N, Kinukawa T, Sugiyama S, Inui K, Nishihara M. Test–retest reliability of prepulse inhibition paradigm using auditory evoked potentials. Neurosci Res 2021;170: 187–194. doi: 10.1016/j.neures.2020.08.011 32987086

[36] Cohen J Statistical power analysis for the behavioral sciences. Hillsdale: Lawrence Erlbaum Associates. 1988.

[37] Herrmann MJ, Sonnek G, Weijers HG, Wiesbeck GA, Böning J, Fallgatter AJ. Electrophysiological indication for a link between serotonergic neurotransmission and personality in alcoholism. Prog Neuropsychopharmacol Biol Psychiatry 2002;26: 157–161. doi: 10.1016/s0278-5846(01)00241-x 11853107

[38] Diedenhofen B, Musch J. Cocor: A comprehensive solution for the statistical comparison of correlations. PLoS One 2015;10: e0121945. doi: 10.1371/journal.pone.0121945 25835001 PMC4383486

[39] Beagley HA, Knight JJ. Changes in auditory evoked response with intensity. J Laryngol Otol 1967;81: 861–873. doi: 10.1017/s0022215100067815 6036752

[40] Picton TW, Woods DL, Baribeau-Braun J, Healey TM. Evoked potential audiometry. J Otolaryngol 1976;6: 90–119. 1030745

[41] Rapin I, Schimmel H, Tourk LM, Krasnegor NA, Pollak C. Evoked responses to clicks and tones of varying intensity in waking adults. Electroencephalogr Clin Neurophysiol 1966;21: 335–344. doi: 10.1016/0013-4694(66)90039-3 4162205

[42] Hari R, Kaila K, Katila T, Tuomisto T, Varpula T. Interstimulus interval dependence of the auditory vertex response and its magnetic counterpart: implications for their neural generation. Electroencephalogr Clin Neurophysiol 1982;54: 561–569. doi: 10.1016/0013-4694(82)90041-4 6181979

[43] Lu ZL, Williamson SJ, Kaufman L. Behavioral lifetime of human auditory sensory memory predicted by physiological measures. Science 1992;258: 1668–1670. doi: 10.1126/science.1455246 1455246

[44] Tanaka E, Inui K, Kida T, Miyazaki T, Takeshima Y, Kakigi R. A transition from unimodal to multimodal activations in four sensory modalities in humans: an electrophysiological study. BMC Neurosci 2008;9: 116. doi: 10.1186/1471-2202-9-116 19061523 PMC2607283

[45] Fujii S, Motomura E, Inui K, Watanabe T, Hakumoto Y, Higuchi K, et al. Weaker prepulse exerts stronger suppression of a change-detecting neural circuit. Neurosci Res 2021;170: 195–200. doi: 10.1016/j.neures.2020.07.007 32702384

[46] Buchwald JS, Rubinstein EH, Schwafel J, Strandburg RJ. Midlatency auditory evoked responses: differential effects of a cholinergic agonist and antagonist. Electroencephalogr Clin Neurophysiol 1991;80: 303–309. doi: 10.1016/0168-5597(91)90114-d 1713841

[47] de Lugt DR, Loewy DH, Campbell KB. The effect of sleep onset on event related potentials with rapid rates of stimulus presentation. Electroencephalogr Clin Neurophysiol 1996;98: 484–492. doi: 10.1016/0013-4694(96)94726-4 8763508

[48] Irimajiri R, Golob EJ, Starr A. Auditory brain-stem, middle- and long-latency evoked potentials in mild cognitive impairment. Clin Neurophysiol 2005;116: 1918–1929. doi: 10.1016/j.clinph.2005.04.010 15998601

[49] Alain C, Chow R, Lu J, Rabi R, Sharma VV, Shen D, et al. Aging enhances neural activity in auditory, visual, and somatosensory cortices: the common cause revisited. J Neurosci 2022;42: 264–275. doi: 10.1523/JNEUROSCI.0864-21.2021 34772740 PMC8802933

