# Supplementary figures and images for: Relationship of loudness-dependent auditory evoked potentials with change-related cortical responses
Source: PLoS One. 2022 Nov 7;17(11):e0277153. doi: 10.1371/journal.pone.0277153 (PMC9639826; doi:10.1371/journal.pone.0277153)

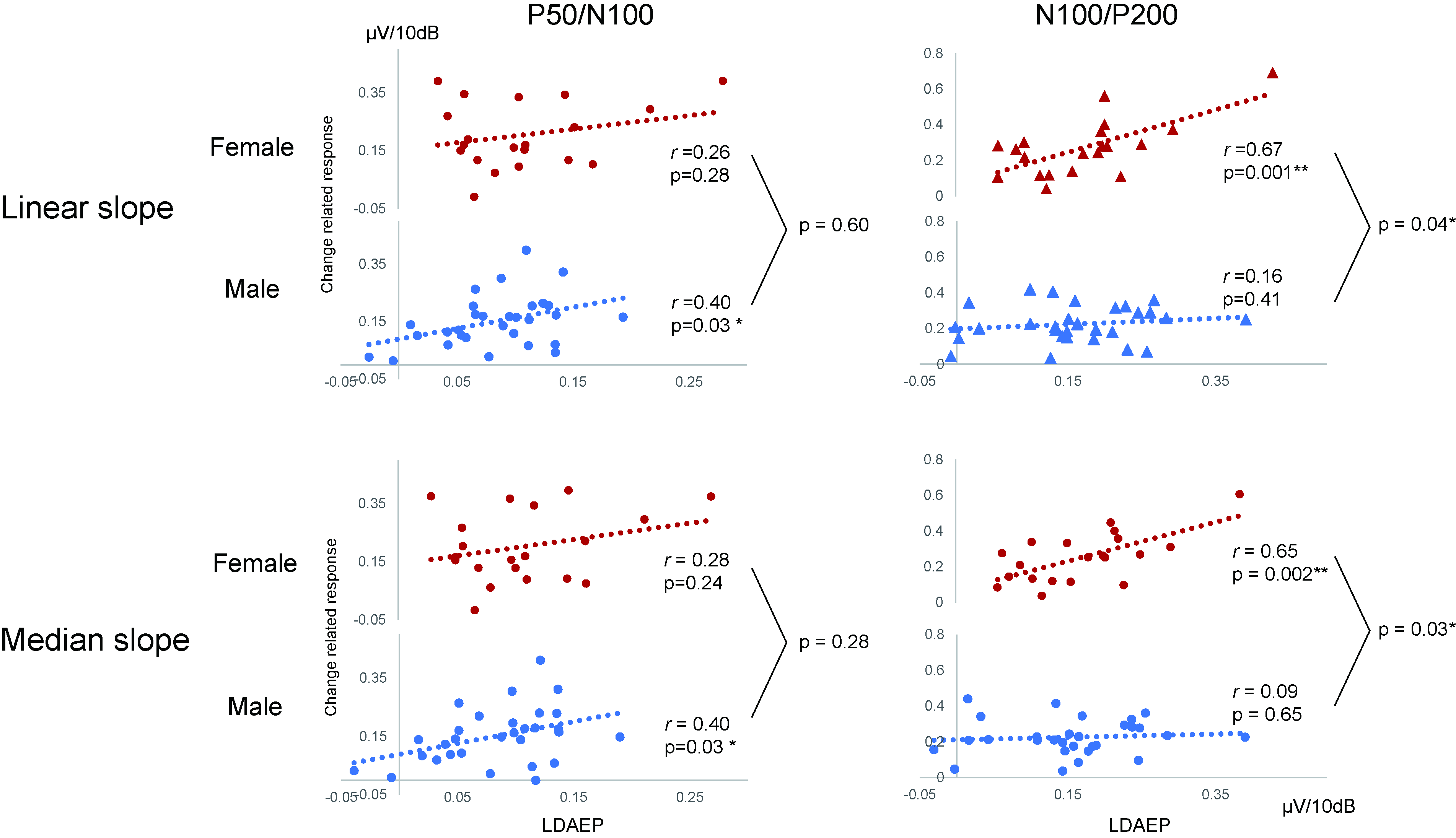

Supplement: S1 Fig — Differences in correlation coefficients between LDAEP and the change-related response by are shown for the P50/N100 and N100/P200 components. (TIF) [file pone.0277153.s001.tif]
